# Mental Health and Well-Being of Migrant Populations in Portugal Two Years after the COVID-19 Pandemic

**DOI:** 10.3390/bs13050422

**Published:** 2023-05-17

**Authors:** Violeta Alarcão, Pedro Candeias, Miodraga Stefanovska-Petkovska, Sónia Pintassilgo, Fernando Luís Machado, Ana Virgolino, Osvaldo Santos

**Affiliations:** 1 Centro de Investigação e Estudos de Sociologia, Iscte—Instituto Universitário de Lisboa, Avenida das Forças Armadas, 1649-026 Lisboa, Portugal; 2 Instituto de Saúde Ambiental, Faculdade de Medicina, Universidade de Lisboa, Avenida Professor Egas Moniz, 1649-028 Lisboa, Portugal; 3 Iscte—Instituto Universitário de Lisboa, Avenida das Forças Armadas, 1649-026 Lisboa, Portugal

**Keywords:** coronavirus disease 2019, depression, anxiety, social determinants of health, migration, resilience, social support

## Abstract

In Portugal, like in other European countries, the COVID-19 pandemic aggravated the risk of poverty and social exclusion faced by migrants. This study aimed to assess mental health and well-being, and their social determinants, among Brazilian and Cape Verdean immigrant populations two years after the COVID-19 pandemic while exploring the role of positive psychological factors such as resilience and perceived social support. We conducted a cross-sectional survey combining online and face-to-face questionnaires for data collection between February and November 2022 on dimensions of mental health considered potentially relevant to the post-pandemic context: psychological distress, anxiety, and depression. Overall, 604 immigrants were included (322 Brazilian and 282 Cape Verdean); 58.5% of those surveyed were women and 41.5% were men. The results revealed that gender (being a woman) was associated with both psychological distress and depression, higher education was associated with anxiety, and that, for the three mental health dimensions under analysis, the perception of discrimination and resilience were negative and positive predictors, respectively. Findings can inform the design and implementation of relevant public mental health promotion programs with a focus on equity targeted to the general population. Such programs would help to address the psychological and social impacts of this long-term, insidious global pandemic that has challenged governments, health care systems, health care professionals, individuals, families, and communities worldwide.

## 1. Introduction

“*In the time of COVID-19 all metrics are not equal when it comes to assessing the pandemic’s unequal effect*”.

*Ferreira H.G. Francisco in* “*Inequality and COVID-19*”

The World Health Organization declared COVID-19, an infectious disease caused by the severe acute respiratory syndrome coronavirus 2 (SARS-CoV-2), a public health emergency of international concern in January 2020 and classified it as a pandemic in March 2020 due to its increasingly global dissemination. The severity of the COVID-19 pandemic’s impact can be well illustrated by three facts: there have been more than 3.1 million deaths related to this disease, 120 million people have been pushed into extreme poverty, and massive global economic challenges have resulted [1]. The pandemic has also had a tremendous impact on migration dynamics across the globe, with almost all countries in the world having implemented coronavirus-related travel restrictions and border shutdowns for managing the outbreak of COVID-19 [2]. As an example, it is estimated that in 2016 around 3.6 billion people travelled short or long distances (excluding train and road transport passengers), and 1 million passengers travelled by plane every day. Through the closing of national borders and the halting of travel worldwide, the pandemic dramatically changed the dynamics of international migration. According to a report by the United Nations, the pandemic may have slowed the growth in the stock of international migrants by around two million by mid-2020, 27 percent less than the growth expected since mid-2019 [3]. Furthermore, the pandemic’s effects on inequality are particularly challenging for the world’s poorest people, with future projections making even more pessimistic estimates. By the end of 2022, at least 75 million more people were expected to have been pushed into poverty (living on less than US$1.90 a day) than was expected before the pandemic [4]. In addition to instigating economic challenges, the COVID-19 crisis has increased the existing inequalities between social groups in health, housing, employment, income, and well-being [4]. The resulting multidimensional inequality particularly affected women, young and older people, people with disabilities, low- and middle-income earners, those with young children, and people from ethnic and racial minority backgrounds. As an illustration, data from the US Centers for Disease Control and Prevention demonstrated that death rates in the United States have been particularly high among Indigenous people [5], while in the United Kingdom (UK) the mortality rate for deaths associated with COVID-19 was the highest among those of black ethnic background and the lowest among those of white ethnic background. The first French large-scale study to explore the issue of social inequalities related to SARS-CoV-2 testing and the dynamics of the pandemic showed that individuals living in the most deprived areas had the highest risk of contracting SARS-CoV-2, but a concomitant lower likelihood of being tested [6,7]. Overall, although the high mortality of certain racial and ethnic groups is of particular concern, data on racial and ethnic inequalities in health is insufficient to give an adequate comprehensive perspective, since much of the available data is aggregated and does not capture differences in family, age, and socio-professional levels [8].

### 1.1. Mental Health Concerns for Migrant Populations

Increasing SARS-CoV-2 infection rates and decreasing human mobility have resulted in a notable decrease in the mental health and well-being of the population worldwide. More specifically, The Global Burden of Disease 2020 study estimated a 27.6% increase in cases of major depressive disorder and a 25.6% increase in cases of anxiety disorders in 2020 [9]. The high levels of stress and deteriorated well-being experienced have resulted in a widespread significant toll on public mental health [10,11,12]. Some studies have compared psychological well-being before and during the pandemic with overall results, demonstrating significant increases in psychopathology [11,13]. For example, the increase in clinically significant psychopathological symptoms was 10% for Germany in 2020 [14], Australia noted twice the prevalence in 2020 compared to non-pandemic circumstances [15], and in the United States, 13.6% of people showed symptoms of serious psychological distress compared to 3.9% in 2018 [16]. Furthermore, the disproportionality of the COVID-19 pandemic towards certain racial and ethnic minority groups is also reflected in its mental health impact. In-depth analyses have demonstrated that the mental health of racial and ethnic minorities such as Blacks, Asians, and Minority Ethnic (BAME) groups has been disproportionately affected by COVID-19 [17,18]. More specifically, although population-representative data indicate that white adults had the highest rates of anxiety and depression before the pandemic, the occurrence of stressful experiences during the pandemic was greater among racial and ethnic minority communities due to the disproportionately greater increases in pandemic-related stressors such as unemployment, food insecurity, infection rates, hospitalizations, and all causes of death among these groups [14,15,16]. Awareness and disparities in access to mental health care among these groups also widened, which led to a “pandemic on a pandemic” [13]. This is deeply concerning and points to the systemically entrenched disadvantages experienced by racial and ethnic minorities and the need to address inequities in these communities to improve overall health outcomes [17,19].

### 1.2. Positive Psychology as a Post-Pandemic Mental Health Remedy

In times of social and economic disturbance, it is noted that the state of an individual’s health often correlates with factors such as resilience and social support [20]. With the prediction that mental health problems will continue to increase in the future, there is a growing recognition of the importance of developing strategies and programs that integrate principles of positive psychology for reducing the prevalence of these problems. Positive psychology practice was introduced almost three decades, or “three waves”, ago [21,22] when psychology was perceived as only a “half-baked” discipline—preoccupied only with the negative side of life and leaving a view of human qualities that is warped and one-sided. Therefore there was a need to start baking the other (“positive”) half [23]. The first wave in positive psychology was essentially characterized by a focus on positive phenomena such as emotions, traits, behaviors, cognitions, and organizations, and thus laid the foundation for people to think more deeply and critically vis-à-vis its foundational notion of the positive. While retaining the focus on these meta-concepts, the second wave added a more nuanced contextual approach to concepts of positive and negative, an appreciation of the ambivalent nature of the good life, and an understanding of the fundamentally dialectical nature of well-being. The third and current wave of positive psychology is broadening the discourse “beyond the individual” and towards complexity in terms of increased interest in super-individual processes and phenomena, becoming more interdisciplinary, multicultural, global, and methodologically richer [21]. It is important to reiterate that the waves are not mutually exclusive; rather, they complement each other [24]. The same relationship holds for COVID-19, as current analyses show. Positive psychology studies what is good about people and what goes right in life [25] by focusing on actual and potential human capacities, and the field increasingly considers that positive psychological factors provide a path toward helping the general public cope with COVID-19 by buffering against mental illness, as well as building and expanding positive practices and new capacities through this crisis [11]. One key protective factor against anxiety and depression, as well as a key determinant of resilience, is adequate social support, which plays a vital role in the reduction in the risk for stress-related mental disorders by buffering the impact of stress in the aftermath of the COVID-19 pandemic [13,19,26]. For instance, a recent study with more than 700,000 college students showed that, during the COVID-19 outbreak, those with low perceived social support were 4.8 and 6.0 times more likely to have symptoms of anxiety and symptoms of depression, respectively, compared to individuals with high perceived social support. Additionally, the same study found that perceived positive social support was a protective factor against the risk for affective disorders, neutralizing the effects of stress and enhancing coping strategies [27]. The findings from this study are consistent with previous investigations which indicate that people with low perceived social support are at a higher risk of psychological pressure, while those with high perceived social support reported reduced levels of stress and anxiety during the COVID-19 pandemic. As an example, Li et al. note that migrants with family-oriented profiles experience lower levels of loneliness and hence more positive mental health outcomes [28].

Hence, psychosocial support from family, friends, schools, and the community may be important to maintain individuals’ psychological well-being and health during the COVID-19 pandemic [29,30].

Portugal has been a destination for immigration for more than three decades, and today it is considered as the second most-favorable citizenship regime in the EU in terms of naturalization rates [31]. However, much like in other countries, the pandemic years in Portugal have been marked by increases in racist and xenophobic phenomena, including towards the Brazilian and Cape Verdean immigrants who make up one of the top five immigrant groups in Portugal [32]. Therefore, it is crucial to understand the social risks among these migrant groups and to identify preventative factors to design effective post-pandemic public health practices and reduce health disparities among racial/ethnic minority groups in the country. 

The present study aimed to contribute to deepening the understanding of mental health and positive psychology in the aftermath of the pandemic by assessing mental health and well-being and their social determinants in the Brazilian and Cape Verdean immigrant populations in Portugal, while exploring the role of positive psychological factors such as resilience and perceived social support. This will support the efforts of the Portuguese public health system to effectively apply their practice to migrant groups who are likely to experience high levels of mental health problems.

## 2. Methods

### 2.1. Study Design and Participants

This paper describes the results of the survey implemented in the EQUALS4COVID19 (Equity in health in times of pandemic) project that aimed to evaluate the mental health and well-being of the Brazilian and Cape Verdean populations in Portugal two years after the COVID-19 pandemic and to understand the social and health care responses during the pandemic.

In Portugal, like in other European countries, the COVID-19 pandemic aggravated the risk of poverty and social exclusion faced by many migrants, who had to struggle not only with a health crisis but also increased economic instability, job, housing and food insecurity, and mobility restrictions [33,34,35,36,37,38]. The immigrant population in Portugal is a heterogeneous group with different nationalities, very diverse sociodemographic characteristics, and different levels of integration [35]. The current study focused on two of the largest immigrant populations in Portugal (nationals of Brazil and Cape Verde), aiming to capture intra-group diversity [30,31,32].

A cross-sectional survey combining self-completion anonymous online computerized and face-to-face structured questionnaires was conducted between February and November 2022. The inclusion criteria for participants were as follows: 18 years of age or older, Brazilian or Cape Verdean nationality, and living in Portugal. The questionnaire was disseminated using different methods (digital social networks, social media, and community institutions, among others), generating a large though non-probabilistic sample. Interviews served to reach individuals with lower digital literacy, lower educational qualifications, and older groups. Questions were as neutral, unbiased, and non-threatening as possible; the survey was anonymous and total confidentiality was guaranteed.

The administered survey was a computerized questionnaire hosted on the Qualtrics software program (Qualtrics, Provo, UT, USA). The questionnaire was pretested among a convenience sample of individuals who met the inclusion criteria to ensure comprehensibility and to solve operational errors.

### 2.2. Measures

The study focused on dimensions of mental health considered potentially relevant to the pandemic context: psychological distress, anxiety, and depression. To assess these outcomes, a set of instruments with sound psychometric properties were used and their internal consistency was calculated with Cronbach’s alpha. All mental health questions were related to the period of the previous two weeks. Participants were also asked about their sociodemographic characteristics and positive-related psychological dimensions, such as resilience and perceived social support.

#### 2.2.1. Psychological Distress

Psychological distress was assessed using the Portuguese version of the five-item Mental Health Inventory (MHI-5) [39].

The MHI-5 is a brief version of the 38-item questionnaire that has been extensively used in research in the last decades to evaluate the same content of its larger version, i.e., the general psychological distress and well-being in general populations [40]. It adopts both a positive and negative point of view in the evaluation of mental health and includes five dimensions, three negative and two positive (e.g., How much of the time have you been a very nervous person? How much of the time have you been a happy person?). The response is given on a six-point scale, and the scoring of the two positive feelings items was done in reverse; thus, the total score ranges between 5 and 30. Following the usual procedure, all scores were converted to fit a range from 0 to 100, with low scores indicating more psychological distress. We used the proposed cut-off of 52 points for psychological distress. In this study, Cronbach’s alpha, which is a measure of internal consistency, was 0.864 for the total sample, 0.893 for the sample of Brazilian immigrants, and 0.807 for the sample of Cape Verdean immigrants.

#### 2.2.2. Anxiety

Anxiety was assessed with a Portuguese version of the Generalised Anxiety Disorder 7-item questionnaire (GAD-7) [41], a self-reported brief scale [42]. The seven items correspond to symptoms of anxiety, based on the criteria of the Diagnostic and Statistical Manual of Mental Disorders, version 4 (DSM-IV), including (1) feeling nervous, anxious, or on edge, (2) not being able to stop or control worrying, (3) worrying too much about different things, (4) trouble relaxing, (5) being so restless that it is hard to sit still, (6) becoming easily annoyed or irritable, and (7) feeling afraid as if something awful might happen. One can answer on a four-point Likert scale ranging from ‘not at all’ to ‘nearly every day’, with a reference period of the past two weeks. The GAD-7 index is obtained by adding the scores from the questionnaire, after having assigned 0 to the least severe situation, 3 to the most severe one, and 1 and 2 to the intermediate ones. The total score between 0 and 21 is dichotomized at the cut-off value of 10+ for case definition. The cut-off points 5, 10, and 15 allow us to classify the anxiety as none/normal (0–4), mild (5–9), moderate (10–14), and severe (15–21) [41]. The Cronbach’s alpha was 0.860 for the total sample, 0.855 for the sample of Brazilian immigrants, and 0.852 for the sample of Cape Verdean immigrants.

#### 2.2.3. Depression

For depression-related symptomatology, the Portuguese-validated version of the Patient Health Questionnaire (PHQ-9) was used [43]. This scale includes nine items, each rated on a 4-point scale from 0 (not at all) to 3 (nearly every day); summed scores range from 0 to 27, with higher scores indicating more severe symptoms of depression. As depression severity categories, we considered the recommendations from Kroenke et al. [44], categorizing scores as follows: 0–4 as minimal, 5–9 as mild, 10–14 as moderate, 15–19 as moderately severe, and 20–27 as severe. The Cronbach’s alpha was 0.887 for the total sample, 0.911 for the sample of Brazilian immigrants, and 0.836 for the sample of Cape Verdean immigrants.

#### 2.2.4. Predictor Variables

Based on an equity-focused literature review on the mental health of migrant populations during the COVID-19 pandemic [45], the following predictor variables were included in the analysis: age, gender, nationality, years in the host country (Portugal), employment status, educational level, and perceived financial situation.

Resilience was measured by the Portuguese version of the 10-item Connor-Davidson Resilience Scale (CD-RISC-10) [46]. The CD-RISC 10 is a unidimensional self-reported scale consisting of 10 items measuring resilience, each rated on a 5-point scale (0–4), with higher scores reflecting greater resilience. Examples of items include “I am able to adapt when changes occur” and “I am not easily discouraged by failure” [26]. In the absence of pre-established cutoff points, a classification system with three categories (low (0–18), medium (19–29), and high (30–40) levels of resilience) was considered based on a k-means cluster analysis for each nationality-based sample group. The Cronbach’s alpha was 0.898 for the total sample, 0.896 for the sample of Brazilian immigrants, and 0.899 for the sample of Cape Verdean immigrants.

Perceived social support was measured using a Portuguese translation of the Brief Form of the Perceived Social Support Questionnaire (F-SozU K-6) [47]. The F-SozU assesses general perceived social support that excludes help from health care professionals. The six items regarding perceived or anticipated social support, and covering generalized experiences rather than concrete situations, are rated on a five-point Likert scale, ranging from one (strongly disagree) to five (strongly agree), with higher scores reflecting higher social support. Examples of items include “I receive a lot of understanding and security from others” and “There is someone very close to me whose help I can always count on”. In the absence of pre-established cutoff points, a classification system with two categories (low level (1–3.33) and high level (3.50–5) of perceived social support) was considered based on a k-means cluster analysis for each group, after computing a mean value for the six items. The Cronbach’s alpha both for the total sample and for the two immigrants’ nationality-based groups was 0.863.

Discrimination was measured using the responses to the question “At some point, during this pandemic, did you feel discriminated against, or did you feel that you received unfair treatment, for being Cape Verdean/Brazilian?”, with the following answer possibilities: “Yes”, “No”, “I don’t know”, and “I prefer not to answer”.

### 2.3. Statistical Analyses

Descriptive statistics [frequency (percent) or mean and standard deviation (SD)], stratified by gender and nationality, were calculated for demographic and socioeconomic variables, mental health outcomes, and positive psychological factors related to mental health-related variables. Bivariate associations between gender and nationality and related variables under study were tested using Chi-Square tests, or one-way ANOVA (with subsequent post-hoc Scheffe or Games–Howell tests, according to the equality or non-equality of the variances), as appropriate. To first identify factors associated with psychological distress, moderate to severe anxiety, and moderate to severe depression by nationality, simple and multiple logistic regression models were used, and odds ratios (OR) were estimated with 95% confidence intervals. For ease of interpretation and possibility of comparison, some of the quantitative variables were converted into dummy variables. Then, we did hierarchical multiple logistic regressions for the total sample to examine the relationship between the independent variables, including nationality. For this, independent variables were categorized into four different blocks: Model 1: sociodemographic variables (nationality, gender, age, educational level); Model 2: sociodemographic variables + social integration indicators (adds length of stay in Portugal and financial situation); Model 3: sociodemographic variables + social integration indicators + discrimination (adds health care access and discrimination); Model 4: sociodemographic variables + social integration indicators + discrimination + positive psychological factors (adds resilience and perceived social support).. For multiple models, Nagelkerke Pseudo R2 is presented as an indicator of the model fit. The data were analyzed using IBM SPSS Statistics version 28 and statistical significance was considered when *p* < 0.05.

### 2.4. Ethical Considerations

The protocol was approved by the appropriate Ethics Committees before the participants’ enrollment and data collection processes. This study has been implemented following the ethical standards laid down in the 1964 Declaration of Helsinki and its later amendments [48]. Participants were informed that they could interrupt their participation at any moment and that their involvement would not require effort besides answering the questions and giving their informed consent before participation. Each participant was assigned a code number to preserve anonymity.

## 3. Results

### 3.1. Sample Characterization and Descriptive Data

The sample was composed of a total of 604 participants (322 Brazilian and 282 Cape Verdean), with 58.5% of those surveyed being women and 41.5% being men. The main characteristics of the study sample are displayed in Table 1. Brazilian immigrant men were younger and had higher employment rates. Compared with Cape Verdean immigrants, Brazilians had a higher educational level (for both genders), had been residing in Portugal for fewer years, and their place of residence was less concentrated in the Lisbon Metropolitan Area. In terms of subjective financial well-being, there were more Cape Verdean (29.9%) than Brazilian (19.0%) individuals indicating that the financial situation of their household was “Difficult or Very difficult”. More than one-third of the participants indicated that their financial situation worsened with the pandemic. Brazilian immigrants reported more difficult access to health care, more discrimination for being an immigrant, and lower levels of resilience compared to the Cape Verdean sample (*p* < 0.001).

### 3.2. Predictors of Mental Distress

#### 3.2.1. Psychological Distress

The prevalence of psychological distress was found to be 28.6% among Brazilian immigrants and 11.7% among Cape Verdean immigrants (*p* < 0.001) (Table 1). Bivariate analysis showed an association between psychological distress and most of the independent variables, except educational level, for both Brazilian and Cape Verdean immigrants (Table 2). In the multiple regressions, psychological distress was found to be associated negatively with the length of stay in Portugal for the Brazilian immigrants (aOR: 0.9; CI: 0.9–1.0), worsening financial situation and impeded access to health care for the Cape Verdean immigrants (aOR: 3.6; CI: 1.3–9.5 and aOR: 2.5; CI: 1.0–6.4, respectively), and self-reporting of discrimination, low levels of resilience, and no perceived social or family support for both of the populations (Table 2).

The four-step hierarchical regression results for psychological distress are presented in Table 3. In Step 1 of the regression model, nationality, gender, and age were found to be associated with psychological distress. The length of stay and financial situation were inserted in the Step 2 regression model, and only gender remained associated and the worsening of the financial situation. In Step 3, these variables remained significant predictors for psychological distress, adding impeded access to health care and self-report of having been discriminated against for being an immigrant. Finally, in Step 4, when resilience and perceived social support were entered as predictors, access to health care lost statistical significance. The final model presented a Nagelkerke’s Pseudo R2 moderate value of 0.31, indicating that 31% of the variation in psychological distress has been explained just by using the covariates included in the model (Table 3).

#### 3.2.2. Anxiety

The prevalence of moderate to severe anxiety was found to be 10.4% among Brazilian immigrants and 5.4% among Cape Verdean immigrants (*p* = 0.002). Multivariate analysis revealed anxiety to be associated with higher education (aOR: 8.9; CI: 1.8–45.0), financial situation (aOR: 0.2; CI: 0.1–0.6), and low levels of resilience (aOR: 5.8; CI: 1.2–28.6) among Brazilian immigrants, while it was associated with the self-reporting of discrimination (aOR: 6.0; CI: 1.1–33.5) and low levels of resilience (aOR: 10.6; CI: 1.0–11.9) among Cape Verdean immigrants (Table 4).

Higher education, financial situation, and low levels of resilience were found to be the major predictors of anxiety for the participating immigrant population, while the other sociodemographic variables, including gender, lost statistical significance in the adjusted models. The final model explained 26% of the variation in the symptoms of anxiety (Table 5).

#### 3.2.3. Depression

The prevalence of symptoms of moderate to severe depression was found to be 29.6% among Brazilian immigrants and 20.7% among Cape Verdean immigrants (*p* < 0.001). Multivariate analysis revealed depression to be associated with being female (aOR: 2.9; CI: 1.4–6.0), being 18–29 years old (aOR: 3.0; CI: 1.0–8.8), low levels of resilience (aOR: 3.4; CI: 1.5–7.8), and no social or family support (aOR: 2.2; CI: 1.0–4.6) among Brazilian immigrants, and with being female (aOR: 4.8; CI: 1.8–12.7), low levels of resilience (aOR: 3.4; CI: 1.3–8.8), and no social or family support (aOR: 4.3; CI: 1.6–11.6) among Cape Verdean immigrants. Reduced rates of depression were observed among Cape Verdean participants aged 30–49 (aOR: 0.1; CI: 0.0–0.6) compared to those 50 years old and over (Table 6).

Self-reported discrimination, low levels of resilience, the absence of perceived social or family support, and gender were found to be the major predictors of depression for the participating immigrant populations, while the other sociodemographic variables, including nationality and age, lost statistical significance in the adjusted models. The final model explained 31% of the variation in the symptoms of depression (Table 7).

## 4. Discussion

This study provides an extension of previous evidence [32,33,49,50,51] by describing mental health and well-being indicators among immigrant populations in Portugal after an already long period of exposure to the pandemic context (more than two years after the first positive case of SARS-CoV-2 in Portugal). Furthermore, we explored the role of positive psychological factors such as resilience and perceived social support in mitigating mental health problems in the post-pandemic context. 

The present study’s findings revealed that gender (being a woman) was associated with both psychological distress and depression, that higher education was associated with anxiety, and that, for the three mental health dimensions under analysis, the perception of discrimination and resilience were negative and positive predictors, respectively. These findings align with previous research studies done prior to the pandemic which suggest that women are more prone to psychological distress, depression, and anxiety compared to their male counterparts [43,44,45]. The available explanations have addressed the biological, psychological, and social risk factors for the gender difference in psychological distress and depression. The group of biological explanations argues based on the genetic differences in chromosomal composition and the possible effect of female hormones. The social risk factors for the gender differences in psychological distress and depression include the difference in social/work roles and expectations for men and women and variations in the social expectations from men and women [52]. It is also known that, in many disadvantaged immigrant minorities established in Western countries, gender relations can be very asymmetric, doubly penalizing women with parental responsibilities or those reconciling work and family life. In the context of the pandemic and confinement, these aspects have become more demanding and have noticeably burdened women more than men in these minorities, which helps explain the higher female incidence of psychological distress and depression [53,54,55]. The psychological risk factors include previous adverse experiences, depression, anxiety, psychological attributes related to vulnerability to life events, and coping skills [53,56,57,58,59,60]. However, it is interesting to note that recent studies conducted during the COVID-19 pandemic have not found gender differences in the experience of depression. For example, recent studies conducted in China during the COVID-19 pandemic found no significant effect of gender, which indicates that male and female participants experienced similar stresses and negative emotions as a result of the pandemic [29,56,57]. On the other hand, a series of studies conducted in the Middle East and Europe highlighted the increased risk of mental health problems among women compared to men living in the same regions [49,50,51]. Similar findings were reported in the Philippines [61]. Although the similarities of findings of this study to the ones done prior to the pandemic indicate the role of gender in mental health as a constant despite changes in the social and public health environment, the differences with the “pandemic period” studies from other regions in the world may point to the potential (and understudied) role of culture in the relationship between gender and mental health.

Regarding the relationship between educational level and anxiety, although this study found that higher education was associated with anxiety, the available knowledge of this relationship is still limited and inconclusive. One longitudinal study suggested that anxiety levels were negatively associated with both the educational level of the respondent’s parents and their educational attainment [62], with another longitudinal study concluding that the protective effect of education accumulated somewhat with time [63]. On the other hand, a cross-sectional study found that low educational levels were significantly associated with anxiety and depression, with coefficients decreasing with increasing age, except for the age group of 65–74 years [63]. Although some studies have suggested that specific mediators explain the relationship between educational level and anxiety [53,55,56,57], the available data remain ambiguous. Without empirical evidence, it can be argued that the perception of and capacity to report anxiety can vary with educational levels, that is, more educated people may be better able to recognize their symptoms than less educated people. If this hypothesis is true, less educated people would not have less anxiety, but would be less able to recognize and report their symptoms because of lower health literacy.

Furthermore, various other factors were found to be associated with both psychological distress and depression among immigrants living in Portugal. This study revealed that they are negatively associated with (a) the length of stay in Portugal, (b) the self-reporting of discrimination, (c) the worsening of the financial situation, (d) difficult access to health care, (e) low levels of resilience, and (f) absence of social or family support. Although these findings are in line with the existing literature mostly predating the pandemic, evidence is still lacking as to the difference in the extent to which they affect the migrant population after the emergence of the pandemic.

Regarding the time residency factor, the results from other studies suggest that immigrants who have spent less time in the receiving country may experience higher levels of distress that diminish in intensity over time [58,59,60]. The link between discrimination, psychological distress, and depression is well documented [64,65,66,67,68,69,70,71]. For example, the study done by Rousseau et al. [70] confirms an increase in the perception of discrimination and psychological distress among Arab Muslim recent immigrant communities after 11 September 2001, while Kim et al. [72] confirm a link between self-reported psychological distress and discrimination among Vietnamese Americans. Other studies have also reported that perceived discrimination is associated with higher levels of psychological pain and mental illness among the Asian American population [64,65,66].

Additionally, this study also found that a poor financial situation is negatively related to psychological distress and depression. Hence, epidemiologic studies using a variety of socio-economic status measures have consistently shown that, among the general population, mortality risk increases as socio-economic status decreases, with a cumulative influence over the individual lifetime [67,68,69]. For example, studies conducted in Portugal concluded that part of the immigrant population is not registered in the social security system, placing them at a specific risk compared to the domestic population [34]. In addition, their financial stress is aggravated by their higher risk of losing their job and suffering a drop in income, which are relatively frequent consequences of ethnic and racial discrimination [51,73,74]. Previous studies have also confirmed the specific link between financial stress and mental health in the migration context [75,76,77,78,79]. Lecerof et al. found that financial difficulties increased the risk of mental health decline [80], while Bruhn et al. found that financial difficulties related to work were the most frequent factor interfering with health care utilization [81]. In line with the findings from this study, other recent studies suggest that access to services also worsened in the opinion of immigrants [82,83]. One study among the immigrant population in Lisbon pointed out that the effect of worsening access to health services during the COVID-19 pandemic is more acute among women and people with lower incomes [83]. Hence, depression and poor mental health outcomes are associated with a higher risk of experiencing barriers to health care, unmet social needs, and poorer economic and mental health outcomes [84]. Interestingly, depression is found not only as an outcome of poor health care access but also as a factor for a greater inability to access health care services, especially among older adults [85,86]. Therefore, countries such as Portugal must address the need to promote the health of the immigrant population residing within the scope of its jurisprudence, regardless of their migrant status or their position in the migratory path [87].

One of the protective factors against poor mental health outcomes among the immigrant population is a high level of resilience [88]. Although this term is still challenging to conceptualize, the literature agrees that migrants with higher resilience scores are expected to have lower levels of psychological distress and depression [89,90]. This is in line with the findings from this study that indicate a negative relationship between resilience and psychological distress and depression. Furthermore, in line with the study conclusions reported here, another study conducted on mental health during the COVID-19 pandemic in Portugal, which surveyed 6079 residents (including 2097 healthcare professionals), demonstrated that resilience has a protective effect on psychological well-being and is associated with a lower risk of anxiety and depression symptoms [91].

Finally, perceived social support was found to have a protective effect on psychological distress and depression by providing a sense of security and belonging, which helps distract individuals from adverse incidents. For example, a study found that perceived social support among Asian Americans moderates against experienced stress from racial discrimination, while perceived emotional support from family also acts as a buffer against stress [71]. Similarly, other studies also found that family support buffered the detrimental effects of racial discrimination concerning the onset of major depressive disorders [92].

### Strengths, Limitations, and Implications of the Research

This study’s results should be interpreted considering several limitations. First, the study utilizes screening tools developed in a single cultural context and based on samples drawn entirely from Western, educated, industrialized, rich, and democratic (WEIRD) societies [93]. Hence, they are biased toward Western ways of thinking and understanding the world. Considering that the findings suggest that members of WEIRD societies are among the least representative populations one could find for generalizing about humans, future research should confront the possibility that culturally specific findings are being misattributed as universal traits [94] and promote the inclusion of non-Western ethno-psychologies, including other forms of cross-cultural [95] and Indigenous psychology [96].

Second, the study uses a structured questionnaire, although it combines online and face-to-face questionnaires for data collection, and therefore relies only on self-reported measures likely subjected to social desirability bias. The sole use of an online survey would have biased the sample’s representativeness, with adults who cannot read, cannot afford a computer/internet, and are less comfortable using a computer being potentially underrepresented.

Third, the study applies a cross-sectional design with data collection during a period of easing government restrictions following prolonged restrictive measures (including lockdown). Thus, the levels of depression, anxiety, and subjective distress of COVID-19 reported by our study should not be considered within a long-term context as we could not monitor changes over time. Moreover, due to the study’s cross-sectional design, we cannot evaluate changes at the population level in terms of the levels of psychological distress, anxiety, and depression associated with pandemic prevention measures and their social and economic consequences. However, given the use of additional measures concerning mental health and its contributing factors, we could shed light on the effects of the pandemic, identifying the most vulnerable groups while informing strategies and actions for improving well-being and reducing health disparities.

Tackling the increased mental health burden due to the COVID-19 pandemic presents challenges to the under-resourced and disorganized mental health-care services in most countries, but also presents opportunities for promoting mental health and targeting the social determinants of poor mental health [9].

## 5. Conclusions

In conclusion, our findings can be used to inform the design and implementation of relevant public mental health promotion programs with an equity focus targeted to the general population. Such programs would help to address the psychological and social impacts of this long-term, insidious global pandemic that has challenged governments, health care systems, health care professionals, individuals, families, and communities worldwide. Furthermore, considering the complex nature of the relationship between these variables and the need for culturally sensitive instruments (preferably longitudinal), further studies could contribute to the more successful interweaving of positive psychological factors with existing and/or new public interventions and programs that have proven to be successful.

## Figures and Tables

**Table 1:** Sociodemographic, health care, and mental health and well-being-related characteristics.

	Brazilian Immigrant Women (BIW) (*n* = 204)	Brazilian Immigrant Men (BIM) (*n* = 116)	Brazilian Immigrants Total (*N* = 322) *	Cape Verdean Immigrant Women (CVIW) (*n* = 148)	Cape Verdean Immigrant Men (CVIM) (*n* = 134)	Cape Verdean Immigrants Total (*N* = 282)	*p* Value
Sociodemographic characteristics							
Age (years), mean	36.89	34.92	36.13	41.39	40.93	41.17	0.001 ^a^
Age group (years), %							
18–29	34.3	43.1	37.6	36.5	32.8	34.8	<0.001
30–49	48.5	44.0	46.9	32.4	34.3	33.3	
50 and over	17.2	12.9	15.5	31.1	32.8	31.9	
Educational level, %							
Until secondary	41.7	47.4	43.8	83.8	83.6	83.7	<0.001
Higher education	58.3	52.6	56.2	16.2	16.4	16.3	
Occupation status, %							
Employed	64.2	74.1	67.7	52.7	64.2	58.2	0.020
Unemployed	9.3	5.2	8.1	8.8	6.7	7.8	
Student	16.7	18.1	17.1	23.0	17.9	20.6	
Inactive	9.8	2.6	7.1	15.5	11.2	13.5	
Place of residence (NUTS II), %							
Norte and Centro	18.7	13.9	16.9	3.4	9.0	6.0	<0.001
Área Metropolitana de Lisboa	75.4	80.9	77.5	95.3	88.8	92.2	
Alentejo and Algarve	5.9	5.2	5.6	1.4	2.2	1.8	
Length of stay in Portugal (mean years)	6.07	6.52	6.21	20.81	21.51	21.16	<0.001 ^b^
Subjective financial well-being (%)							
Comfortable or Very comfortable	27.3	38.1	31.0	19.7	27.4	23.4	0.021
Enough for my needs	48.6	40.0	45.5	47.4	41.9	44.8	
Difficult or Very difficult	19.1	18.1	19.0	31.4	28.2	29.9	
I’d rather not answer	4.9	3.8	4.5	1.5	2.4	1.9	
Financial situation worsened with the pandemic (%)	44.3	34.3	41.0	42.3	33.1	37.9	0.136
Health care and mental health and well-being-related variables							
Access to health care (%)							
Easy access to health care	53.6	53.8	53.6	75.4	76.9	76.1	<0.001
Hard access to health care	46.4	46.2	46.4	24.6	23.1	23.9	
Perception of discrimination for being an immigrant, %							
Didn’t feel discrimination	52.2	56.6	53.4	74.6	79.2	76.8	<0.001
Felt discrimination	47.8	43.4	46.6	25.4	20.8	23.2	
Resilience, %							
High level of resilience	31.1	45.5	36.1	40.7	60.6	50.0	<0.001
Low/Middle level of resilience	68.9	54.5	63.9	59.3	39.4	50.0	
Perceived social/family support, %							
High perceived social support	77.2	84.3	79.9	81.8	87.4	84.4	0.123
Low perceived social support	22.8	15.7	20.1	18.2	12.6	15.6	
Mental Health Inventory (MHI-5)							
Without psychological distress	67.6	79,3	71.4	83.1	94.0	88.3	<0.001
With psychological distress	32.4	20,7	28.6	16.9	6.0	11.7	
Generalized Anxiety Disorder (GAD-7)							
None/mild anxiety	86.6	94.7	89.6	91.9	97.7	94.6	0.002
Moderate/severe anxiety	13.4	5.3	10.4	8.1	2.3	5.4	
Patient Health Questionnaire (PHQ-9)							
Minimum/mild depression	64.3	82.3	70.4	72.3	87.1	79.3	<0.001
Moderate/severe depression	35.7	17.7	29.6	27.7	12.9	20.7	

* Two participants selected the option “other” in gender; ^a^ Equal variances not assumed, post-hoc Games–Howell (BIW = BIM), (BIW = CVIW), (BIW = CVIM), (BIM ≠ CVIW), (BIM ≠ CVIM), (MCV = CVIM); ^b^ Equal variances not assumed, post-hoc Games–Howell (BIW = BIM) (BIW ≠ CVIW) (BIW ≠ CVIM) (BIM ≠ CVIW) (BIM ≠ CVIM) (CVIW = CVIM).

**Table 2:** Prevalence of psychological distress and associated factors.

	Brazilian Immigrants			Cape Verdean Immigrants		
	Psychological Distress % (95%CI)	Crude OR * (95%CI)	Adjusted OR ** (95%CI)	Psychological Distress % (95%CI)	Crude OR * (95%CI)	Adjusted OR ** (95%CI)
Gender						
Women	32.4 (26.2–39.0)	1.7 (1.0–2.9)	1.8 (0.9–3.6)	16.9	3.2 (1.4–7.4)	2.3 (0.8–6.4)
Men	20.7 (14.1–28.7)	1.0	1.0	6.0	1.0	1.0
Age group (years)						
18–29	32.2 (24.4–40.9)	2.5 (1.1–5.8)	2.7 (0.8–8.8)	14.3	1.1 (0.5–2.5)	0.6 (0.1–2.7)
30–49	29.8 (22.9–37.4)	2.2 (1.0–5.1)	2.9 (0.9–9.1)	7.4	0.5 (0.2–1.4)	0.5 (0.1–1.8)
50 and over	16.0 (7.9–27.9)	1.0	1.0	13.3	1.0	1.0
Educational level						
Until secondary	29.1 (22.1–36.9)	1.0	1.0	11.0	1.0	1.0
Higher education	28.2 (22.0–35.0)	1.0 (0.6–1.6)	0.9 (0.4–1.8)	15.2	1.4 (0.6–3.6)	1.7 (0.5–6.0)
Occupation status						
Employed	29.4 (23.6–35.6)	4.4 (1.0–19.2)	-	9.1	0.4 (0.1–1.0)	-
Unemployed	42.3 (25.0–61.3)	7.7 (1.5–39.9)	-	13.6	0.6 (0.1–2.5)	-
Student	27.3 (16.9–40.0)	3.9 (0.8–18.9)	-	12.1	0.5 (0.2–1.6)	-
Inactive	8.7 (1.9–25.1)	1.0	-	21.1	1.0	-
Length of stay in Portugal	-	0.9 (0.9–1.0)	0.9 (0.9–1.0)	-	1.0 (1.0–1.0)	1.0 (0.9–1.0)
Financial well-being						
Enough for my needs, Comfortable or Very comfortable	23.8 (18.7–29.6)	1.0	-	12.8	1.0	1.0
Difficult or Very difficult	38.2 (26.2–51.4)	2.0 (1.1–3.7)	-	21.8	3.4 (1.6–7.2)	-
Financial situation has worsened	37.8 (29.5–46.7)	2.6 (1.5–4.5)	1.7 (0.9–3.3)	21.2	4.1 (1.8–9.1)	3.6 (1.3–9.5)
Access to health care						
Easy access to health care	18.7 (13.2–25.4)	1.0	1.0	8.1	1.0	1.0
Hard access to health care	36.6 (28.8–44.9)	2.5 (1.5–4.3)	1.2 (0.6–2.3)	25.8	3.9 (1.8–8.5)	2.5 (1.0–6.4)
Discrimination for being an immigrant						
Didn’t feel discrimination	17.5 (11.6–24.8)	1.0	1.0	24.6	1.0	1.0
Felt discrimination	36.8 (29.0–45.1)	2.7 (1.5–4.9)	2.3 (1.1–4.6)	9.3	3.2 (1.5–7.0)	2.8 (1.0–7.5)
Resilience						
High level of resilience	10.9 (6.1–17.7)	1.0	1.0	6.6	1.0	1.0
Low/Middle level of resilience	37.4 (30.9–44.4)	4.9 (2.5–9.5)	1.9 (0.9–4.1)	16.9	2.9 (1.3–6.5)	2.2 (0.8–6.3)
Perceived social/family support						
High perceived social support	18.4 (13.9–23.7)	1.0	1.0	8.8	1.0	1.0
Low perceived social support	61.7 (49.1–73.2)	4.1 (2.3–7.4)	4.7 (2.2–10.1)	28.6	4.2 (1.8–9.4)	2.9 (1.0–8.5)
Nagelkerke’s Pseudo R2	-	-	0.311	-	-	0.319

The * crude odds ratio (OR), ** multiple adjusted OR, and the respective 95% confidence intervals (95%CI) were computed in logistic regression models. Participants with missing values for any of the independent variables were excluded from the models.

**Table 3:** Hierarchical regression analyses for predictors of psychological distress.

	Model 1: Sociodemographic Variables		Model 2: Sociodemographic Variables + Integration		Model 3: Sociodemographic Variables + Integration + Discrimination		Model 4: Sociodemographic Variables + Integration + Discrimination + Positive Psychological Variables	
	OR * (95%CI)	*p* Value	OR * (95%CI)	*p* Value	OR * (95%CI)	*p* Value	OR * (95%CI)	*p* Value
Nationality, Brazilian	1.9 (1.1–3.3)	0.028	1.5 (0.8–2.7)	0.249	1.2 (0.6–2.3)	0.618	1.1 (0.6–2-2)	0.781
Gender, Women	2.1 (1.3–3.6)	0.003	2.0 (1.2–3.5)	0.007	2.1 (1.2–3.6)	0.007	1.9 (1.1–3-3)	0.029
Age, 18–29	2.0 (1.0–3.8)	0.050	1.7 (0.8–3.7)	0.149	1.8 (0.8–3.9)	0.135	1.4 (0.6–3.2)	0.438
Age, 30–49	1.5 (0.8–2.9)	0.211	1.3 (0.7–2.6)	0.428	1.4(0.7–2.9)	0.321	1.3 (0.6–2.7)	0.563
Higher education	1.2 (0.7–2.1)	0.446	1.2 (0.7–2.1)	0.517	1.1(0.7–2.0)	0.656	1.1 (0.6–1.9)	0.853
Length of stay			1.0 (1.0–1.0)	0.201	1.0(1.0–1.0)	0.394	1.0 (1.0–1.0)	0.151
Financial situation worsened with the pandemic			2.8 (1.7–4.5)	<0.001	2.4 (1.4–3.9)	0.001	2.1 (1.3–3.6)	0.005
Hard access to health care					1.7 (1.0–2.8)	0.045	1.5 (0.9–2.6)	0.126
Felt discriminated against for being an immigrant					2.4(1.4–4.0)	0.001	2.5 (1.5–4.4)	0.001
Low perceived social support							4.2 (2.3–7.6)	<0.001
Low/Middle level of resilience							2.0 (1.1–3.7)	0.023
Nagelkerke’s Pseudo R2	0.090		0.155		0.211		0.309	

The * multiple adjusted odds ratio (OR) and the respective 95% confidence intervals (95%CI) were computed in logistic regression models. Participants with missing values for any of the independent variables were excluded from the models.

**Table 4:** Prevalence of anxiety (moderate to severe) and associated factors.

	Brazilian Immigrants			Cape Verdean Immigrants		
	Anxiety % (95%CI)	Crude OR * (95%CI)	Adjusted OR ** (95%CI)	Anxiety % (95%CI)	Crude OR * (95%CI)	Adjusted OR ** (95%CI)
Gender						
Women	13.4 (9.2–18.6)	2.8 (1.1–7.1)	3.2 (0.8–12.2)	8.1 (4.5–13.3)	3.8 (1.0–13.8)	1.5 (0.3–8.0)
Men	5.3 (2.2–10.5)	1.0	1.0	2.3 (0.6–5.9)	1.0	1.0
Age group (years)						
18–29	12.5 (7.5–19.3)	3.4 (0.8–15.6)	4.7 (0.5–43.6)	8.2 (4.0–15.0)	1.9 (0.6–6.7)	0.2 (0.0–2.0)
30–49	10.8 (6.6–16.6)	2.9 (0.6–13.1)	2.7 (0.3–23.2)	3.2 (0.9–8.4)	0.7 (0.2–3.3)	0.1 (0.0–1.5)
50 and over	4.0 (0.8–12.2)	1.0	1.0	4.4 (1.5–10.2)	1.0	1.0
Educational level						
Until secondary	5.7 (2.7–10.5)	1.0	1.0	5.1 (2.8–8.5)	1.0	1.0
Higher education	14.0 (9.5–19.7)	2.7 (1.2–6.2)	8.9 (1.8–45.0)	6.5 (1.9–16.4)	1.3 (0.3–4.8)	1.9 (0.3–13.7)
Occupation status						
Employed	10.7 (7.1–15.4)	1.3 (0.3–5.7)	-	1.8 (0.5–4.8)	0.2 (0.0–1.1)	-
Unemployed	7.7 (1.6–22.5)	0.9 (0.1–6.8)	-	9.1 (1.9–26.1)	1.2 (0.2–7.6)	-
Student	10.9 (4.7–21.1)	1.3 (0.2–6.9)	-	12.3 (5.7–22.6)	1.6 (0.4–6.8)	-
Inactive	8.7 (1.9–25.1)	1.0	-	7.9 (2.3–19.6)	1.0	-
Length of stay in Portugal	-	0.9 (0.8–1.0)	1.0 (0.9–1.0)	-	1.0 (0.9–1.0)	1.0 (0.9–1.0)
Financial well-being						
Enough for my needs, Comfortable or Very comfortable	11.1 (7.5–15.5)	1.0	-	4.4 (2.1–8.1)	1.0	-
Difficult or Very difficult	3.6 (0.8–11.2)	0.3 (0.1–1.3)	-	7.7 (3.3–15.2)	1.8 (0.6–5.4)	-
Financial situation has worsened	5.9 (2.7–11.2)	0.4 (0.2–1.1)	0.2 (0.1–0.6)	6.1 (2.6–12.1)	1.2 (0.4–3.7)	0.6 (0.1–3.1)
Access to health care						
Easy access to health care	7.7 (4.3–12.7)	1.0	1.0	3.6 (1.6–6.8)	1.0	1.0
Hard access to health care	11.9 (7.3–18.2	1.6 (0.7–3.6)	1.7 (0.6–4.9)	11.3 (5.2–20.9)	3.5 (1.2–10.3)	2.4 (0.5–11.7)
Discrimination for being an immigrant						
Felt discrimination	9.6 (5.5–15.3)	1.4 (0.6–3.3)	1.0 (0.3–2.9)	8.2 (3.2–17.0)	2.0 (0.6–6.5)	6.0 (1.1–33.5)
Didn’t feel discrimination	7.1 (3.6–12.6)	1.0	1.0	4.3 (2.0–8.3)	1.0	1.0
Resilience						
High level of resilience	2.7 (0.8–7.1)	1.0	1.0	2.9 (1.0–6.8)	1.0	1.0
Low/Middle level of resilience	13.8 (9.5–19.2)	5.7 (1.7–19.4)	5.8 (1.2–28.6)	7.4 (3.8–12.7)	2.6 (0.8–8.6)	10.6 (1.0–11.9)
Perceived social/family support						
High perceived social support	7.9 (5.0–11.9)	1.0	1.0	4.4 2.3–7.6	1.0	1.0
Low perceived social support	16.7 (8.9–27.6)	2.3 (1.0–5.3)	1.7 (0.5–5.4)	9.5 3.3–21.1	2.3 (0.7–7.7)	2.8 (0.4–17.4)
Nagelkerke’s Pseudo R2	-	-	0.332	-	-	0.293

The * crude odds ratio (OR), ** multiple adjusted OR, and the respective 95% confidence intervals (95%CI) were computed in logistic regression models. Participants with missing values for any of the independent variables were excluded from the models.

**Table 5:** Hierarchical regression analyses for predictors of anxiety (moderate to severe).

	Model 1: Sociodemographic Variables		Model 2: Sociodemographic Variables + Integration		Model 3: Sociodemographic Variables + Integration + Discrimination		Model 4: Sociodemographic Variables + Integration + Discrimination + Positive Psychological Variables	
	OR * (95%CI)	*p* Value	OR * (95%CI)	*p* Value	OR * (95%CI)	*p* Value	OR * (95%CI)	*p* Value
Nationality, Brazilian	0.8 (0.3–2.2)	0.732	0.8 (0.2–2.4)	0.638	0.6 (0.2–2.0)	0.430	0.6 (0.2–2.0)	0.399
Gender, Women	2.8 (1.1–7.2)	0.033	2.9 (1.1–7.5)	0.027	2.8 (1.1–7.3)	0.034	2.4 (0.9–6.3)	0.084
Age, 18–29	2.0 (0.7–6.1)	0.203	1.6 (0.5–5.4)	0.412	1.6 (0.5–5.4)	0.463	1.0 (0.3–3.6)	0.986
Age, 30–49	1.0 (0.3–3.2)	0.960	1.0 (0.3–3.1)	0.956	1.0 (0.3–3.1)	0.961	0.7 (0.2–2.3)	0.518
Higher education	4.9 (1.9–12-7)	0.001	4.6 (1.7–12.4)	0.002	4.5 (1.7–12.1)	0.003	4.1(1.5–11.3)	0.006
Length of stay			1.0 (0.9–1.0)	0.560	1.0 (0.9–1.0)	0.748	1.0 (0.9–1.0)	0.582
Financial situation worsened			0.4 (0.2–1.0)	0.050	0.3 (0.1–0.8)	0.021	0.3 (0.1–0.7)	0.008
Hard access to health care					2.0 (0.8–4.6)	0.118	1.8 (0.8–4.3)	0.174
Felt discrimination					2.0 (0.8–4.7)	0.128	1.8 (0.8–4.4)	0.184
Low perceived social support							1.8 (0.7–4.4)	0.219
Low/Middle level of resilience							5.8 (1.6–20.8)	0.007
Nagelkerke’s Pseudo R2	0.129		0.154		0.188		0.259	

The * multiple adjusted odds ratio (OR) and the respective 95% confidence intervals (95%CI) were computed in logistic regression models. Participants with missing values for any of the independent variables were excluded from the models.

**Table 6:** Prevalence of symptoms of depression (moderate to severe) and associated factors.

	Brazilian Immigrants			Cape Verdean Immigrants		
	Depressive Symptoms % (95%IC)	Crude OR * (95%CI)	Adjusted OR ** (95%CI)	Depressive Symptoms % (95%IC)	Crude OR * (95%CI)	Adjusted OR ** (95%CI)
Gender						
Women	35.7	2.3 (1.4–4.1)	2.9 (1.4–6.0)	27.7	2.6 (1.4–4.8)	4.8 (1.8–12.7)
Men	17.7	1.0	1.0	12.9	1.0	1.0
Age group (years)						
18–29	38.1	3.7 (1.5–8.9)	3.0 (1.0–8.8)	32.0	1.8 (0.9–3.4)	1.9 (0.5–6.7)
30–49	27.8	2.3 (1.0–5.6)	1.5 (0.5–4.3)	8.6	0.4 (0.1–0.9)	0.1 (0.0–0.6)
50 and over	14.3	1.0	1.0	21.1	1.0	1.0
Educational level						
Until secondary	31.9	1.0	1.0	20.5	1.0	1.0
Higher education	27.7	0.8 (0.5–1.3)	0.9 (0.5–1.8)	21.7	1.1 (0.5–2.3)	1.3 (0.4–4.6)
Occupation status						
Employed	26.9	1.2 (0.4–3.4)	-	12.9	0.3 (0.1–0.6)	-
Unemployed	44.0	2.5 (0.7–9.0)	-	31.8	0.9 (0.3–2.8)	-
Student	35.8	1.8 (0.6–5.7)	-	29.8	0.8 (0.3–2.0)	-
Inactive	23.8	1.0	-	34.2	1.0	-
Length of stay in Portugal	-	0.9 (0.9–1.0)	1.0 (0.9–1.0)	-	1.0 (1.0–1.0)	1.0 (1.0–1.0)
Financial well-being						
Enough for my needs, Comfortable or Very comfortable	30.7	1.0	-	16.2	1.0	-
Difficult or Very difficult	34.5	1.4 (0.7–2.6)	-	28.2	2.0 (1.1–3.8)	-
Financial situation has worsened	36.1	1.8 (1.1–3.0)	1.3 (0.7–2.4)	25.3	1.7 (0.9–3.1)	1.7 (0.7–4.3)
Access to health care						
Easy access to health care	23.9	1.0	1.0	16.8	1.0	1.0
Hard access to health care	34.3	1.7 (1.0–2.8)		30.6	2.2 (1.1–4.2)	1.5 (0.6–3.9)
Discrimination for being immigrant						
Didn’t feel discrimination	20.6	1.0	1.0	17.9	1.0	1.0
Felt discrimination	37.5	2.3 (1.3–4.0)	1.7 (0.9–3.2)	29.5	1.9 (1.0–3.8)	2.2 (0.9–5.8)
Resilience						
High level of resilience	10.9	1.0	1.0	10.3	1.0	1.0
Low/Middle level of resilience	40.0	5.4 (2.8–10.6)	3.4 (1.5–7.8)	30.1	3.8 (1.9–7.3)	3.4 (1.3–8.8)
Perceived social/family support						
High perceived social support	23.0	1.0	1.0	14.9	1.0	1.0
Low perceived social support	55.0	4.1 (2.3–7.4)	2.2 (1.0–4.6)	50.0	5.7 (2.8–11.6)	4.3 (1.6–11.6)
Nagelkerke’s Pseudo R2	-	-	0.285	-	-	0.403

The * crude odds ratio (OR), ** multiple adjusted OR, and the respective 95% confidence intervals (95%CI) were computed in logistic regression models. Participants with missing values for any of the independent variables were excluded from the models.

**Table 7:** Hierarchical regression analyses for predictors of depressive symptoms (moderate to severe).

	Model 1: Sociodemographic Variables		Model 2: Sociodemographic Variables + Integration		Model 3: Sociodemographic Variables + Integration + Discrimination		Model 4: Sociodemographic Variables + Integration + Discrimination + Positive Psychological Variables	
	OR * (95%CI)	*p* Value	OR * (95%CI)	*p* Value	OR * (95%CI)	*p* Value	OR * (95%CI)	*p* Value
Nationality, Brazilian	1.3 (0.8–2-2)	0.343	1.2 (0.7–2.2)	0.571	1.0 (0.6–1.9)	0.900	0.9 (0.5–1.8)	0.871
Gender, Women	3.5 (2.1–5.9)	<0.001	3.4 (2.0–5.8)	<0.001	3.5 (2.1–6.0)	<0.001	3.2 (1.8–5.5)	<0.001
Age, 18–29	2.6 (1.4–4.8)	0.002	2.7 (1.3–5.4)	0.006	2.7 (1.3–5.6)	0.006	2.0 (0.9–4.2)	0.083
Age, 30–49	0.8 (0.4–1.5)	0.488	0.8 (0.4–1.5)	0.423	0.8 (0.4–1.5)	0.467	0.6 (0.3–1.2)	0.155
Higher education	1.2 (0.7–2.0)	0.521	1.2 (0.7–2.1)	0.495	1.1 (0.7–1.9)	0.644	1.1 (0.6–1.9)	0.774
Length of stay			1.0 (1.0–1.0)	0.849	1.0(1.0–1.0)	0.902	1.0 (1.0–1.0)	0.660
Financial situation has worsened			2.0 (1.2–3.2)	0.004	1.7 (1.0–2.8)	0.032	1.4 (0.9–2.4)	0.169
Hard access to health care					1.5 (0.9–2.4)	0.143	1.3 (0.8–2.2)	0.320
Felt discrimination					1.9 (1.1–3.1)	0.014	1.9 (1.1–3.2)	0.020
Low perceived social support							3.1 (1.7–5.5)	<0.001
Low/Middle level of resilience							3.1 (1.7–5.7)	<0.001
Nagelkerke’s Pseudo R2	0.152		0.177		0.207		0.307	

The * multiple adjusted odds ratio (OR) and the respective 95% confidence intervals (95%CI) were computed in logistic regression models. Participants with missing values for any of the independent variables were excluded from the models.

## Data Availability

The raw data supporting the conclusions of this article will be made available by the authors upon request.
